# Biocide-resistant *Pseudomonas oleovorans* isolated from water-based coatings used in construction

**DOI:** 10.1093/jimb/kuaf015

**Published:** 2025-06-17

**Authors:** Muatasem Latif Ali, Lionel Ferrieres, Tuulia Hyötyläinen, Jana Jass

**Affiliations:** MTM Research Centre, School of Science and Technology, Örebro University, Örebro, Sweden; Saint-Gobain Sweden AB, Scanspac, Glanshammar, Sweden; Saint-Gobain Research Paris, Aubervilliers, France; MTM Research Centre, School of Science and Technology, Örebro University, Örebro, Sweden; The Life Science Centre - Biology, School of Science and Technology, Örebro University, Örebro, Sweden

**Keywords:** *Pseudomonas oleovorans*, biocide resistance, 5-chloro-2-methyl-isothiazolin-3-one (CMIT), 2-methylisothiazolin-3(2H)-one (MIT), 1,2-Benzisothiazolin-3(2H)-one (BIT)

## Abstract

Biocides are crucial in industrial applications to minimize microbial growth and prevent product spoilage. Water-based construction coatings are susceptible to microbial contamination during manufacturing and storage and this adversely impacts product properties, reduces shelf-life, and leads to substantial commercial losses. The future trend to lower the biocide concentrations in water-based coatings raises concerns about the emergence of biocide-resistant microbes. This study aims to identify and characterize the biocide-resistant microbe isolated from construction water-based coating materials to better understand its mechanisms of resistance. A total of 63 samples were collected from spoiled products, raw materials, and water from a manufacturing facility, and *Pseudomonas oleovorans* P4A were identified in all biocides-treated samples. A comparison between a *P. oleovorans* reference strain, 1045, and the P4A isolate revealed distinct colony morphology, growth rate and sensitivity to biocides and antibiotics. The P4A isolate was threefold more resistant to 5-chloro-2-methyl-isothiazolin-3-one and 1.5-fold more resistant to benzothiazolinone (BIT) compared to the reference strain. Conversely, it was 1.4-fold more sensitive to methylisothiazolinone (MIT) compared to the reference strain. No cross-resistance to antibiotics was observed. Metabolomic analysis using liquid chromatography combined with high-resolution mass spectrometry of lipids and polar metabolites showed that P4A had a relatively higher amount of lipids, while 1045 had a relatively higher amount of polar metabolites identified. A significant difference in lipid composition, specifically in diacylglycerol, phosphatidic acid, phosphatidylcholine, and phosphatidylserine was observed between *P. oleovorans* strains 1045 and P4A. These distinctions highlight increased lipid metabolism in *P. oleovorans* P4A and this may contribute to its adaptation to biocides. Microbial resistance can directly affect the effectiveness of these products, leading to an increased need for frequent maintenance and replacement, safety concerns, and environmental implications.

**One-Sentence Summary**: Biocide-resistant *Pseudomonas oleovorans* isolate exhibited reduced growth rate and increased lipid levels relative to the reference strain.

## Graphical Abstract

**Graphical Abstract:**
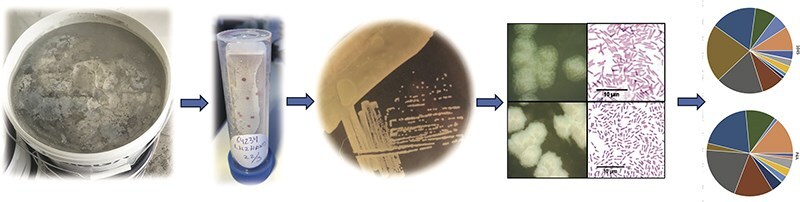

## Introduction

Microbial spoilage of water-based coating materials results in substantial commercial losses, amounting to multimillion-dollar damage annually (Liu et al., 2022). The high-water content, together with organic substances, create a favorable environment for microbial growth during storage, thus reducing product quality and shelf-life. In several European countries, building companies are legally obliged to provide customers with products that are free from defects and resistant to microbial contamination. Thus to mitigate microbial growth, isothiazolones such as 5-chloro-2-methyl-4-isothiazolin-3-one (CMIT), 2-methyl-4-isothiazolin-3-one (MIT), and 1,2-benzisothiazolin-3-one (BIT) are commonly used in coating materials due to their antimicrobial activity (Collier et al., 1990). However, isothiazolones are strong sensitizers producing skin irritations, and allergies and may pose eco-toxicological hazards, therefore their use has been restricted by EU legislation (Silva et al., 2020). CMIT and MIT are usually combined in a 3:1 proportion and used in combination with BIT to preserve different types of coating material products. This is one of the most cost-effective biocide combinations for preserving coating material products in the wet state (Williams, 2006).

Water-based coatings used in construction contain non-sterile raw materials, such as minerals, which can serve as reservoirs for bacteria, yeast, and fungi. The presence of these microorganisms can lead to product spoilage and reduced shelf-life, compromising quality and performance (Tusher et al., 2023). As microbes grow, they change the properties of the product so that it becomes more suitable for the growth of a greater diversity of microbes. While a significant portion of research has focused on microbial contamination within the paint industry, there is a noticeable gap in research regarding microbial contamination of other water-based coating materials, as highlighted by Rosa and colleagues (Rosa et al., 2008). Continuous microbiological monitoring is both expensive and time-consuming, leading to a relative lack of comprehensive studies in this area (Knight et al., 2018). To enhance manufacturing efficiency, multiple products frequently share production lines and equipment. Raw materials are usually sourced from non-sterile environments to mitigate production costs and introduce a significant level of microbiological contamination from the initial production stages. Consequently, the application of biocides becomes imperative to safeguard products against microbial contamination and spoilage (Stoveken et al., 2005). Understanding microbial contamination levels and the mechanisms through which microbes compromise industrial products may lead to enhanced preservation strategies, ideally minimizing reliance on biocides, which may reduce cost and environmental impact. Developing alternative preservation strategies would address challenges related to antimicrobial resistance, a common issue encountered in manufacturing processes (Lambert, 2005).

*Pseudomonas* species are one of the most common bacteria isolated from industrial processes due to their ability to degrade a wide array of organic substances and their resistance to a broad range of antimicrobial compounds (Abdel-Malek et al., 2002). *Pseudomonas* spp. also have cellular properties that contribute to their resistance to different biocides (Verdial et al., 2023). Together, these properties allow them to respond to their surroundings and enable them to survive stress. Resistance mechanisms in *Pseudomonas* spp. are usually related to a combination of strategies that the microbe uses to survive in toxic environments (Li et al., 2015). Gram-negative bacteria have an outer membrane that provides a barrier to some biocides and prevents them from entering the cells (Chapman, 2003). Thus, low permeability and structural integrity of the cell wall due to the lipopolysaccharides and other membrane lipids provide pseudomonads with high intrinsic resistance to biocides (Maillard, 2018). In addition, membrane functions associated with energy production, cell signaling and managing stress response contribute to biocide resistance (Santos & Preta, 2018). The other major contributors to biocide resistance are gene mutations (Lambert, 2005). The *Pseudomonas* genus also exhibits the capacity to form biofilms, which are complex accumulations of microorganisms embedded in an exopolymer matrix typically affixed to surfaces, and this protects them from biocides (Wingender & Flemming, 2011). Genes that can confer biocide resistance can co-exist with antibiotic resistance genes, both on the bacterial chromosomes and transferable plasmids (Pal et al., 2015). Several studies describe biocide resistance in *Pseudomonas* spp., but little research has been done on physiological changes associated with the resistance phenotype (Chapman, 2003; Vijayakumar et al., 2016).

Isothiazolone are non-oxidizing biocides that are used to control microbial growth and biofilm formation (Williams, 2006). Unlike other biocides, they diffuse across the microbial cell membrane and react with the nucleophilic groups of cellular components. They can, for example, react with the thiols from cysteines of active protein sites, blocking their enzymatic activity and produce free radiacals, thus potentially causing cell damage or death (Fabrega et al., 2009). Resistance to isothiazolones has been observed in a number of bacteria including *P. aeruginosa*, where there the resistant strain lacked the protease, outer membrane protein T (Brözel & Cloete, 1994; Sondossi et al., 1999). We recently reported that sub-MIC levels of isothiazolone influence metabolic changes in *P. oleovorans* during exposure and these may contribute to the increased biocide resistance (Ali et al., 2024). This suggests that certain adaptations or alterations in cellular membrane properties and metabolism would contribute to isothiazolone resistance in bacteria.

In the present study, spoiled water-based construction coating materials were evaluated for microbial contamination. The only microbe consistently isolated and able to persist in the water-based coating product in the presence of biocides was identified as *P. oleovorans*. Since it was the only species found in all the spoiled samples containing biocide, it was suspected to be the most problematic contaminant. The isolate, *P. oleovorans* P4A, was therefore characterized to better understand the mechanisms of persistence in biocide-containing products. To that aim, the morphological, cultural and biochemical properties, with a focus on metabolomic profiles, were compared between the industrial isolate and a reference strain, *P. oleovorans* 1045, to characterize the potential differences between their sensitivity to three biocides, namely, CMIT, MIT, and BIT, and seven antibiotics.

## Material and Methods

### Sample Collection and Microbial Isolation

**Fig. 1.**
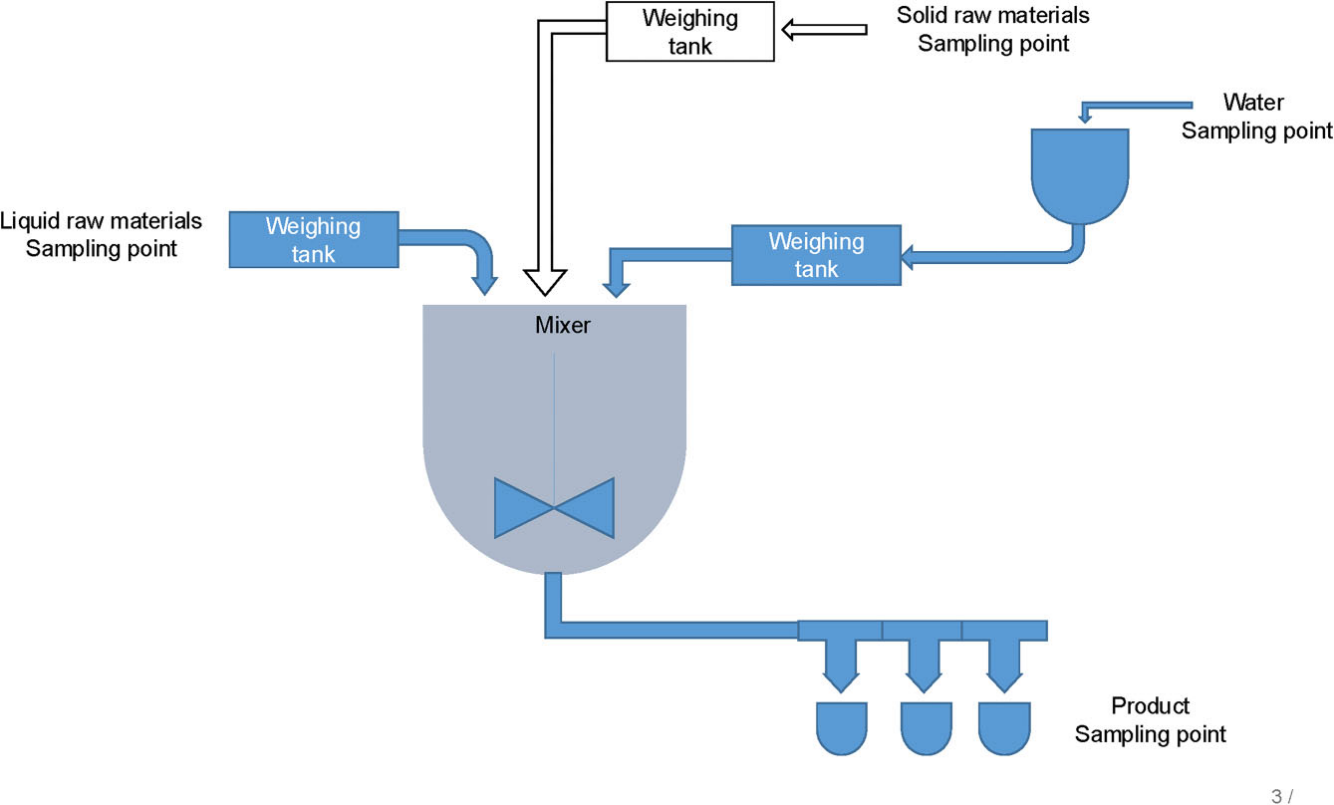
Schematic diagram of the process flow and sampling points.

A total of 63 different samples were collected from spoiled products and raw materials at different stages of production from a single industrial site. Figure 1 represents a typical production plant and sampling points. The distribution and number of samples collected from spoiled contaminated industrial products, biocide-treated samples, raw materials (both liquid and solid phases), and biocide-free industrial products are detailed in Table 1. A total of 33 contaminated industrial products, each from a distinct production batch (33 batches), along with 22 different types of raw materials and two municipal water samples used in production, were collected and analyzed. Each sample consisted of a minimum of three replicates. As controls, six distinct batches of industrial product without biocides were produced and sampled. Three of these biocide-free batches were prepared in the laboratory using the same formulation as in production. The remaining three samples were extracted from the production line before the addition of biocides. Samples were transported from the production site to the laboratory in sterile containers (RS PRO 250 ml HDPE Wide Neck Storage Bottles) at ambient temperature. Semi-quantitative analysis of microbial contamination was done using Mikrocount combi dip slides (Schülke & Mayr GmbH, Germany) according to manufacturers recommendations. Briefly, samples were carefully applied to both sides of a dip slide using a sterile cotton swab, ensuring even distribution, and incubated at 30 °C (±2) for 48 h (±2 h). Each slide contained nutrient TTC (triphenyltetrazolium chloride) and Rose Bengal media for the detection of bacterial growth and yeast and fungal growth on either side of the slide (contact area of 10 cm²). Single colonies from the dip slides were transferred to Luria–Bertani (LB, Difco^TM^, Becton, Dickinson and Co., USA) agar plates and individual isolates were stored at −80 °C.

**Table 1.** The sample types, the number of the samples and microbiological identification

Samples	Number of samples	Microbe identification
Spoiled water-based coating	33	*Pseudomonas oleovorans*
Different types of raw materials	22	*Pseudomonas oleovorans* *Pseudomonas stutzeri* *Bacillus jeotgali* *Bacillus subterraneus Glutamicibacter arilaitensis* *Paenibacillus amylolyticus* *Bacillus licheniformis*
Water-based coating without biocide	6	*Micrococcus luteus* *Yarrowia lipolytica* *Pseudomonas oleovorans*
Water samples	2	*Pseudomonas fluorescens* *Pseudomonas tolaasii*

Identification was done by using MALDI-TOF MS. a Bruker MALDI Biotyper.

Microbiological contamination was quantified by viable counts on LB agar. Briefly, samples were serially diluted in phosphate-buffered saline (PBS; pH 7.4 137 mM NaCl, 2.7 mM KCl, 10 mM Na_2_HPO_4_, and 1.8 mM KH_2_PO_4_) and 50 µl of the dilutions were spread onto LB agar. After incubation for 48 h at 30 °C, the colony-forming units (CFUs) were determined. Three colonies from each plate were isolated by streaking onto a new agar plate for microbiological identification and subsequent analyses.

### Microbial Identification

Isolated colonies were identified using Matrix-Assisted Laser Desorption/Ionization Time-of-Flight mass spectrometry (MALDI-TOF MS; BD Bruker MALDI Biotyper) with tof-user@FLEX-PC and software version 4.3.18 (Bruker Daltonics, Bremen, Germany). Each microbe was analyzed multiple times to ensure accuracy and precision of the identification. The *P. oleovorans* P4A isolate was sent to Eurofins Genomics (Germany) for sequencing and 16S rRNA analysis for species identification.

### Growth Rate Determination

*Pseudomonas oleovorans* P4A was isolated from contaminated water-based coating material and *P. oleovorans* 1045 (CCUG 2087, NBCIB 6576, and DSM 1045) was purchased from CCUG (Culture Collection University of Gothenburg) as a reference organism. Bacteria were cultured on LB agar for 48 h at 30 °C to ensure optimum growth conditions. Three to five colonies were transferred to 10 ml brain heart infusion (BHI) broth and incubated overnight in an orbital shaker at 150 rpm and 30 °C. The bacterial inoculum was adjusted to an optical density (OD) of between 0.08 and 0.1 with BHI broth (equivalent to 1.5 × 10^8^ CFU/ml). Growth was monitored every hour until 48 h using a spectrophotometer at 600 nm wavelength (Infinite F50 Microplate, Tecan). Three biological replicates and three technical replicates for each were done to determine the growth curves and the maximum generation time (G) for both strains. The CFUs were determined for both bacterial strains by counting individual colonies after serial dilution and spread on a plate. The exponential growth rate for each bacterial culture was determined according to the formula *G* = *t*/*n*, where *G* is generation time, *t* is time in h, and *n* is the number of generations.

### Biocide Sensitivity Assay

The minimum inhibitory concentration (MIC) and minimum bactericidal concentration (MBC) of 1,2-Benzisothiazolin-3(2H)-one (BIT, CAS-No. 2634–33-5, purity (GC) >98.0%, TCI EUROPE N.V., Netherlands), 5-Chloro-2-methyl-4-isothiazolin-3-one (CMIT, CAS-No. 26172–55-4, purity (GC) >98.0%, LGC Standards GmbH, Germany) and 2-Methyl-4-isothiazolin-3-one (MIT, CAS-No. 2682–20-4, purity (GC) >98.0%, Sigma–Aldrich Inc. USA) were assessed for both *P. oleovorans* P4A and 1045. The biocides were dissolved in 10% dimethyl sulfoxide (DMSO, purity ≥99%, Merck Life Science AB) in distilled water and subsequently diluted in BHI broth to produce working stock solutions of 0.02 mg/ml CIT, 0.24 mg/ml MIT and 0.41 mg/ml BIT and a final DMSO concentration of <0.03% v/v.

Inoculum preparation was done according to a standard procedure (Collier et al., 1990). Briefly a colony from an overnight culture was transferred to 5 ml of BHI and incubated overnight at 30 °C in a shaker at 150 rpm until it reached turbidity equal to, or greater than the OD of a 0.5 McFarland standard. For each strain, three biological replicates were prepared. The cultures were centrifuged at 5,000 × *g* for 5 min (Himac CT6E), the pellets were washed twice with PBS and resuspended in BHI to the OD of 0.5 McFarland standard, which was approximately 1 × 10^8^ cfu/ml.

The MIC assay was performed in a flat bottom 96-well polystyrene microtiter plate. To each well, a 100 μl of BHI media, 50 μl biocide diluted in BHI media and 50 μl of inoculum adjusted to the 0.5 McFarland standard were added. The final biocide concentration in each well containing 200 μl is specified in Supplementary Table S1. For each biocide concentration, 8 technical replicates and 4 biological replicates were conducted, and the results were reported in terms of mg/l. The microtiter plate was incubated at 30 °C for 48 h and the MIC was determined as the lowest concentration with no visible growth. The MBC was determined by spotting 10 µl onto LB agar plates from concentrations representing the MIC, two concentrations higher than MIC and one concentration lower than MIC of the three biocides (BIT, MIT, and CMIT) and incubating at 30 °C. Three replicates were conducted for each concentration. Observations were recorded at 24-h and 48-h intervals to monitor bacterial growth, and the colony-forming units per millilitre (cfu/ml) were calculated. The MBC was determined by the lowest concentration that inhibited bacterial growth (Rodriguez-Melcon et al., 2021).

### Antimicrobial Susceptibility Testing

Antimicrobial susceptibility was assessed for the two bacteria, *P. oleovorans* P4A and 1045, using the ETEST^®^ (https://www.biomerieux-diagnostics.com/etestr) according to the manufacturer’s instructions. Seven different antibiotics from 4 classes: β-Lactams (Imipenem, Meropenem, Ceftazidime), Aminoglycosides (Amikacin, Tobramycin), Fluoroquinolone (Ciprofloxacin), and polymyxin B (Colistin). Briefly, a bacterial suspension at 0.5 McFarland standard was spread onto a Mueller–Hinton Agar Plate (MH) (BD Difco™ Mueller–Hinton Broth) and the antibiotic test strip was placed onto the agar surface. The plates were incubated overnight at 30 °C and the MIC values were interpreted according to EUCAST breakpoint values (version 14.0) (EUCAST, 2024).

### Bacterial Metabolomic Analyses

Cultures were prepared for the metabolomic analysis by inoculating 5 ml of BHI medium with *P. oleovorans* P4A and 1045 and incubating at 30 °C while shaking at 150 rpm for 24 h. Eight separate cultures were prepared for each strain. The cultures were adjusted to an optical density (OD_600_) of 1 and incubated for a further 24 h. Four replicate tubes were prepared for each strain. Cultures were harvested by centrifugation at 6,000 rpm for 10 min and the pellet was transferred to a new microfuge tube. After gentle resuspension in PBS, the bacterial pellets were vortexed, centrifuged again, and the saline was removed. The bacterial pellets were frozen and stored at −80 °C. Two methods were used for analysis of lipids and polar and semipolar metabolites by ultra-high-performance liquid chromatography quadrupole time-of-flight mass spectrometry (UHPLC-QTOFMS) equipped with dual ESI ionization (Agilent Technologies).

Lipidomic analyses were done using a modified Folch procedure (Folch et al., 1957) as previously described (Ali et al., 2024). Briefly, the bacterial cell samples were weighed, and PBS was added to achieve a ratio of 1 mg of bacterial mass to 10 µl of buffer. A volume of 20 µl of the cell homogenate was extracted with 150 µl of chloroform:methanol (2:1) containing a 2.5 ppm internal standard solution. The internal standard solution consisting of 1,2-diheptadecanoyl-sn-glycero-3-phosphoethanolamine (PE [17:0/17:0]), N-heptadecanoyl-D-erythro-sphingosylphosphorylcholine (SM [d18:1/17:0]), N-heptadecanoyl-D-erythro-sphingosine (Cer [d18:1/17:0]), 1,2-diheptadecanoyl-sn-glycero-3-phosphocholine (PC [17:0/17:0]), 1-heptadecanoyl-2-hydroxy-sn-glycero-3-phosphocholine (LPC [17:0]) and 1-palmitoyl-d31-2-oleoyl-sn-glycero-3-phosphocholine (PC [16:0/d31/18:1]) were purchased from Avanti Polar Lipids, Inc. (Alabaster, AL, USA), and triheptadecanoylglycerol (TG [17:0/17:0/17:0]) was purchased from Larodan AB (Solna, Sweden) (Supplementary Table S2). Following extraction, the samples were incubated on ice for 30 min, centrifuged and 60 µl from the lower phase of each sample was transferred to a glass vial with an insert. Subsequently, 60 µl of CHCl_3_: CH_3_OH (2:1. v/v) was added to each sample and they were stored at −80 °C until further analysis.

For the analysis of polar and semipolar metabolites, 80 µl of the homogenized cell samples were extracted with 180 µl of acetonitrile (ACN) containing internal standards (13C-labeled PFOS, PFOA, PFDA, PFHxS, and PFUnDA as well as Valine-d8, Glutamic acid-d5, Succinic acid-d4, Heptadecanoic acid, Lactic acid-d3, Citric acid-d4, 3-Hydroxybutyric acid-d4, Arginine-d7, Tryptophan-d5, Glutamine-d5, CA-d4, LCA-d4, UDCA-d4, CDCA-d4, DCA-d4, GCA-d4, GLCA-d4, GUDCA-d4, and GCDCA-d4). After vortexing and 3 min of ultrasonication, the samples were centrifuged for 5 min at 7,800 rpm. A volume of 180 µl was collected and evaporated to dryness, and resuspended in 40 µl of 70% methanol in water.

Calibration curves were generated using standard compounds both for lipids and other metabolites and the samples were analyzed using ultra-high-performance liquid chromatography quadrupole time-of-flight mass spectrometry (UHPLC-QTOF MS) with Agilent Technologies equipment (Supplementary Table S3). The UHPLC system utilized in this study was a 1290 Infinity II system and the mass spectrometer was a 6545 QTOF. Data acquisition was performed using MassHunter B.06.01 software (Agilent Technologies) and quality control measures were consistently applied throughout the dataset (Nygren et al., 2011), using pooled samples, in-house quality control samples and blank samples. Identification was performed using an in-house spectral library with m/z, tandem mass spectrometry (MS/MS), and retention time data.

### Statistical Analyses

A two-sample independent *t*-test was used to compare lipid and metabolite concentrations between *P. oleovorans* P4A and 1045 strains. Significance was established with a *P*-value ≤0.05. Metabolomics data analysis utilized MetaboAnalyst 6.0 software (Chong et al., 2018), with data filtering to retain only lipids and metabolites detected in over 70% of samples for robustness. A two-step pre-processing approach involving logarithmic transformation and auto-scaling was applied to normalize and enhance the data for subsequent statistical analyses.

Principal component analysis (PCA) was employed for exploratory data analysis, reducing potentially correlated variables into uncorrelated principal components that captured the most variance. Univariate analysis methods identified significantly different lipids and metabolites between the isolate and reference strains. Hierarchical clustering, combined with heatmap analysis, was used to produce a visual representation of a data table, with concentration values. For two-group data, FC analysis *t*-tests and volcano plots were used, with FC analysis calculating mean concentration ratios, *t*-tests assessing statistical significance and volcano plots integrating both methods for feature selection based on biological significance or p-value considering significance when *P* < 0.05.

## Results

### Microbial Diversity in Starting Material and Water-Based Coatings

The culturable microbial diversity in the different starting materials and final products, including those containing biocides, was determined from one manufacturing site of water-based coatings (Fig. 1). The 63 samples originated from coating materials containing biocides, sourced from spoiled industrial products and different raw starting materials that were in either dry solid, liquid, or semi-solid phases, including municipal water used in production. As a reference, products without biocides were included in the study. The raw materials had a range of microorganisms, encompassing different *Pseudomonas* and *Bacillus* species, in addition to *Glutamicibacter arilaitensis* and *Paenibacillus amylolyticus* (Table 1). Among these, only *P. oleovorans* was consistently identified across all biocide-treated spoiled industrial products. Furthermore, *P. oleovorans* was not found in the municipal water used in production, suggesting that the contaminating source to likely be the raw materials.

**Fig. 2.**
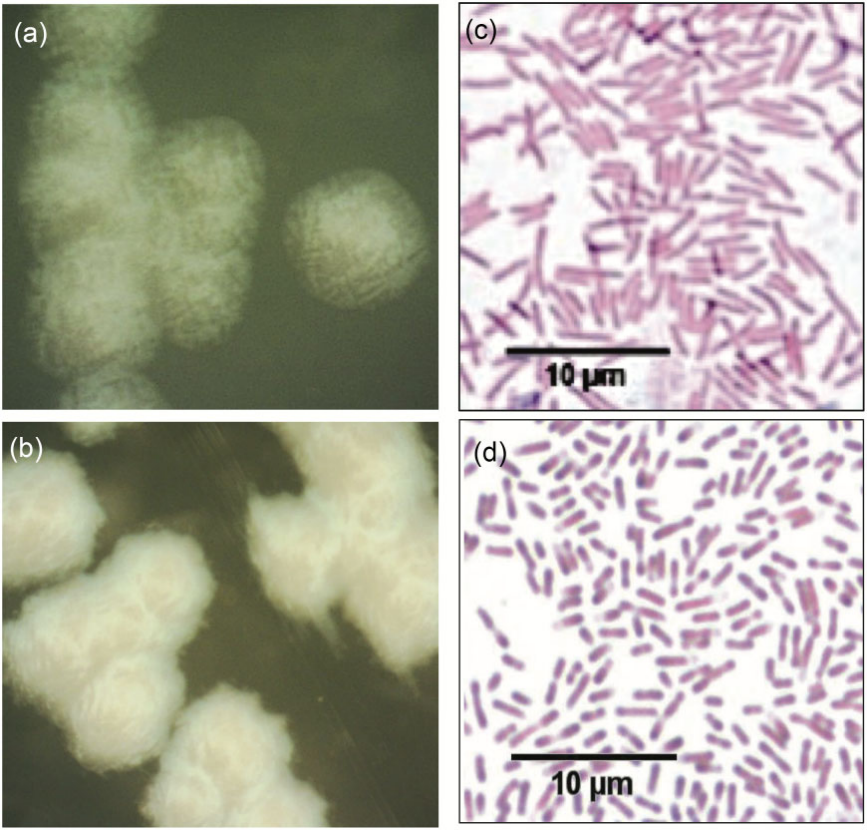
Colony and cell morphology of (a, c) *P. oleovorans* P4A and (b, d) 1045. Cultures were grown on LB agar and analyzed at early stationary phase. *Pseudomonas oleovorans* P4A cells appeared longer than *P. oleovorans* 1045 cells. Gram staining micrographs of (c) *P. oleovorans* P4A and (d) 1045 were taken using 100× objective on an Olympus BX 51 Fluorescent Microscope.

*Pseudomonas oleovorans* P4A isolated from the spoiled water-based coating material was identified using MALDI-TOF MS and confirmed by 16S rRNA sequencing (Supplementary Fig. S1). To identify properties that could be associated with biocide resistance, the isolate was compared to an evolutionarily related reference strain, *P. oleovorans* 1045, isolated from industrial cutting fluid in the USA (Poehlein et al., 2017). Despite belonging to the same species, the two strains exhibited different colony characteristics when grown on LB agar (Fig. 2). *Pseudomonas oleovorans* P4A colonies were smooth, circular and transparent (Fig. 2a), whereas 1045 colonies were irregular, opaque, and slimy (Fig. 2b). The cell structure also differed between the two strains, where *P. oleovorans* P4A produced slightly longer rods than 1045 (Fig. 2c and d). The different characteristics may be due to adaptation of the present isolate (P4A) to the biocide and coating environment.

### Characterization of the Growth Rate of the Isolated *P. oleovorans*

**Fig. 3.**
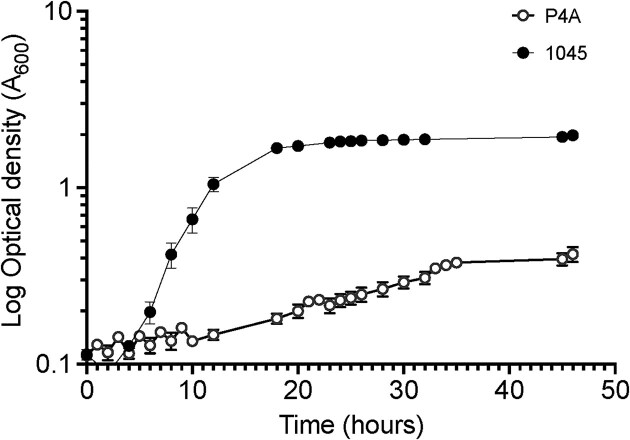
Growth curves for *P. oleovorans* P4A and 1045. Bacteria were grown in BHI medium and incubated at 30 °C and shaking at 150 rpm. Each point is an average of 4 samples and the error bars are SD.

Growth rates were compared between *P. oleovorans* P4A and the refence strain, since slower growth has been associated with increased antimicrobial resistance in bacteria. The exponential growth rate in BHI at 30 °C was significantly different between the two strains (Fig. 3). *Pseudomonas oleovorans* 1045 grew more rapidly with a growth rate of 0.31 ± 0.01 generations/hour and a generation time of 2.3 h, reaching stationary phase by 18 h. In contrast, the growth rate and generation time for the *P. oleovorans* P4A strain were notably slower, with a growth rate of 0.06 ± 0.004 generations/hour, a generation time of 12.5 h and reaching stationary phase after 35 h. The total viable count at stationary phase was 3.0 × 10^8^ ± 1.0 × 10^7^ (SD) cfu/ml for *P. oleovorans* P4A after 48 h and 1.83 × 10^8^ ± 3.21 × 10^7^ (SD) cfu/ml for *P. oleovorans* 1045 after 24 h, confirming the difference in growth.

### Antimicrobial Resistance of *P. oleovorans*

Isothiazolones such as CMIT, BIT, and MIT, are commonly employed biocides in industrial applications for the preservation of coating materials. *P. oleovorans* P4A was consistently isolated from spoiled coating materials containing those biocides and therefore was tested for biocide sensitivity. The MIC is the level of biocide that inhibits bacterial growth but may not kill the bacteria, whereas the MBC is the level that kills the bacteria. Comparative analysis of the MIC revealed that *P. oleovorans* P4A exhibited a threefold higher MIC value for CMIT and a 1.5-fold higher MIC value for BIT than the reference strain *P. oleovorans* 1045 (Table 2). Interestingly, *P. oleovorans* P4A displayed a slightly lower MIC value for MIT than *P. oleovorans* 1045, indicating increased sensitivity to this biocide compared to the reference strain. The MBC for *P. oleovorans* P4A were higher than those for *P. oleovorans* 1045 in the case of CMIT and BIT (Table 2). In contrast, the MBC for MIT for *P. oleovorans* P4A was lower than that for *P. oleovorans* 1045. This suggests varying susceptibility of *P. oleovorans* P4A to the different isothiazolone preservation biocides, with distinct sensitivity patterns observed in comparison to the reference strain.

**Table 2.** Susceptibility of *P. oleovorans* P4A and 1045 to 3 isothiazolone biocides

Biocides	*Pseudomonas oleovorans* P4A		*Pseudomonas oleovorans* 1045		Max dosage permitted by regulations^1^
Types^2^	MIC^3^ (mg/l)	MBC (mg/l)	MIC^3^ (mg/l)	MBC (mg/l)	(mg/l)
**CMIT**	12 ± 1	14	5 ± 1	6	15
**MIT**	13 ± 3	30	26 ± 9	40	15
**BIT**	925 ± 50	1000	667 ± 58	800	500

1 https://echa.europa.eu/sv/regulations/biocidal-products-regulation/product-types.

2 CMIT, 5-chloro-; 5-chloro-2-methyl-isothiazolin-3-one, 2-methyl-isothiazolin-3-one; BIT, Benzothiazolinone and MIT, Methylisothiazolinone.

3 MIC values are average ± standard deviation of 4–6 replicates. All MIC are statistically significant between bacterial strains using *t*-test analysis with *P* < 0.01.

To determine if there was cross-resistance with antibioticis, we assessed the susceptibility of *P. oleovorans* P4A and the reference strain *P. oleovorans* 1045, to seven different antibiotics spanning various classes. Neither strain was resistant to any antibiotics tested, however, comparative analysis showed that the MIC values for imipenem and meropenem were higher in *P. oleovorans* P4A compared to *P. oleovorans* 1045. Conversely, MIC values were lower for ceftazidime, colistin, amikacin, tobramycin, and ciprofloxacin in P4A (Table 3). The antibiotic susceptibility testing underscored significant variations in MIC values between the two *P. oleovorans* strains.

**Table 3.** Antibiotic sensitivity of *P. oleovorans* 1045 and P4A

Antibiotic*	MIC mg/l^†^			
	*S* ≤	*R* >	P4A	1045
Imipenem (IP)	0.001	4	2 (S)	1.5 (S)
Meropenem (MP)	2	8	2 (S)	0.125 (S)
Ceftazidime (TZ)	0.001	8	0.75	1.5 (S)
Amikacin (AK)	16	16	1.5 (S)	4 (S)
Tobramycin (TM)	2	2	0.25 (S)	0.5 (S)
Ciprofloxacin (CI)	0.001	0.5	0.047 (S)	0.094 (S)
Colistin (CO)	4	4	0.25 (S)	1.5 (S)

† The MIC values considered sensitive (*S*) or resistant (*R*) are based on EUCAST breakpoint values V.14.

* Antibiotics Class: β-Lactams (Imipenem—IP, Meropenem—MP, Ceftazidime—TZ), Aminoglycosides (Amikacin—AK, Tobramycin -TM), Fluoroquinolone (Ciprofloxacin—CI), and polymyxin (Colistin—CO).

### Metabolomic Analysis of *P. oleovorans* P4A and 1045

**Fig. 4.**
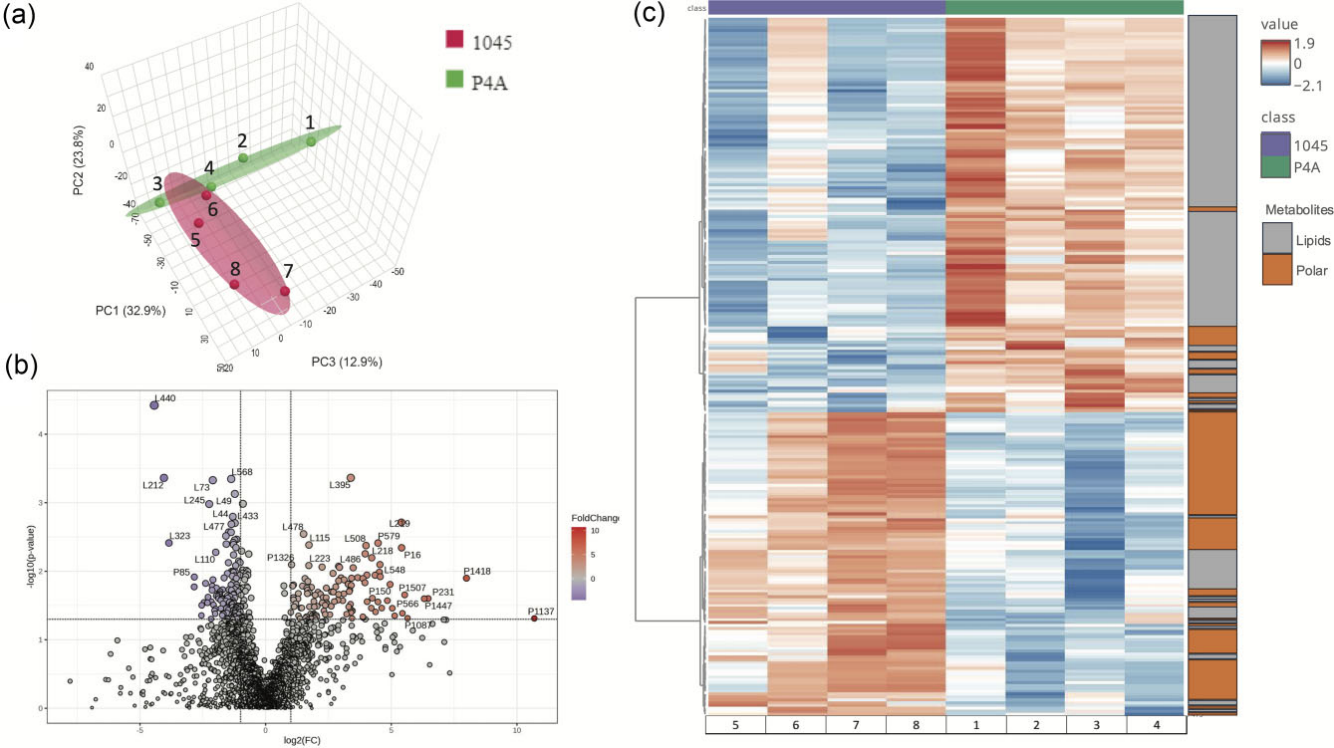
The metabolite analysis of *P. oleovorans* P4A and 1045. (a) Synchronized 3D plot PCA of the metabolites in P4A and 1045 sample groups. (b) Volcano Plot displaying the significant variations in total of 244 metabolites (Lipids and polar) in *P. oleovorans* P4A and *P. oleovorans* 1045 strains (*P* < 0.05). (c) A Heatmap featuring 244 significantly altered metabolites (*t*-test. *P* < 0.05).

Based on the colony appearance and that isothiazolones need to difuse into the cell for biocide activity, it is likely that alterations to the cell membrane would contribute to biocide resistance. Therefore, comparative metabolomic analysis of lipids and polar metabolites was conducted on *P. oleovorans* P4A and 1045 grown aerobically in BHI broth for 24 h at 30 °C. A total of 2237 metabolites were identified, and of these 2181 metabolites were unknown, while 56 were known metabolites. Of the total, only 244 metabolites (10.9%) were statistically different at a *P*-value of 0.05, while the remaining 1,993 metabolites were not. The PCA of the metabolic profiles from the two strains showed distinct differences, where 69.6 % of the difference could be explained by the first three dimensions (Fig. 4a). The variation in the lipid metabolites between strains is illustrated by the Volcano plot (Fig. 4b), where fold change (FC) is integrated with the *t*-test. A heatmap of the concentrations of the 244 significantly different metabolites (*P* < 0.05) using Wards clustering algorithm showed that there was a distinct difference in the levels of both lipids and polar metabolites between the two strains (Fig. 4c). A notable feature was that *P. oleovorans* P4A had predominantly higher levels of lipid metabolites, and lower levels of polar metabolites relative to the reference strain 1045. This raises questions about the involvement of lipids in the biocide resistance phenotype (Fig. 4c).

**Fig. 5.**
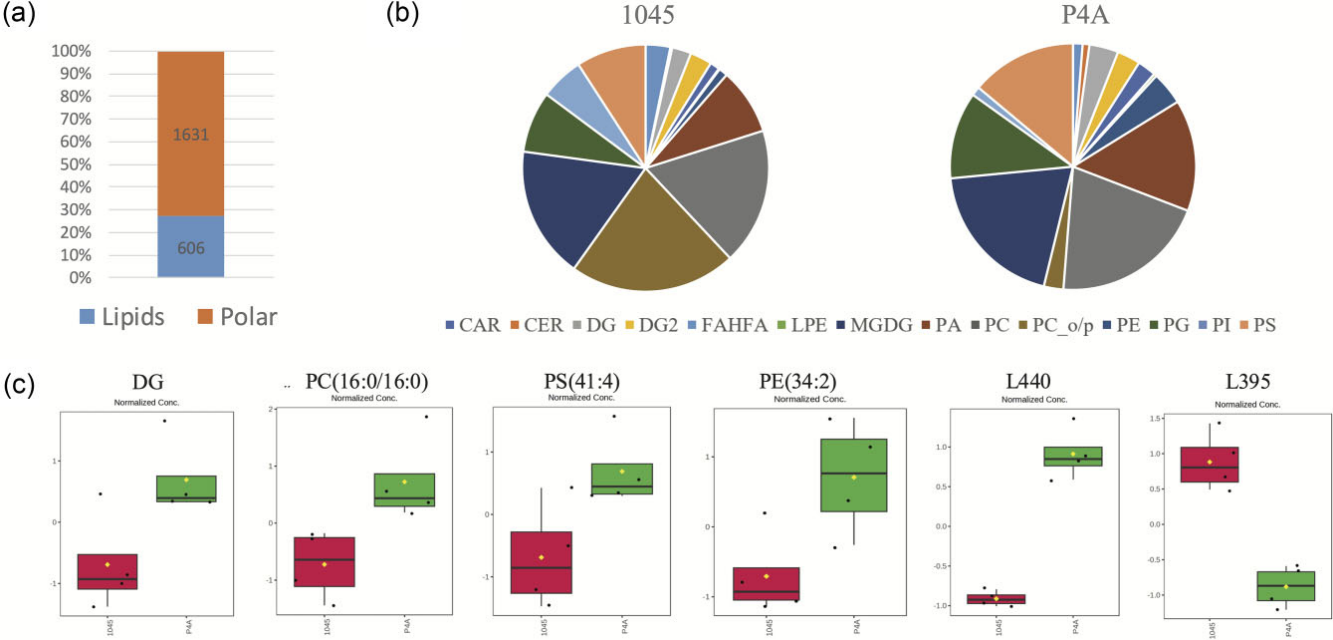
Lipidomic comparison between *P. oleovorans* P4A and 1045. (a) The proportion of lipids and polar metabolites in P4A and 1045. (b) Relative distribution of the levels of identified lipids in P4A and 1045 isolates. The identified lipids include carnitine (CAR), ceramide (CER), diacylglycerol (DG), 1,2-diacylglycerol (DG2), fatty acid hydroxy fatty acid (FAHFA), lysophosphatidylethanolamine (LPE), monogalactosyldiacylglycerol (MGDG), phosphatidic acid (PA), phosphatidylcholine (PC), phosphatidylcholine oleoyl/palmitoyl (PC_o/p), phosphatidylethanolamine (PE), phosphatidylglycerol (PG), phosphatidylinositol (PI), and phosphatidylserine (PS). (c) PCA analysis of metabolic profiles in *P. oleovorans* P4A and 1045 strains. The components view of DG, PC, PS, PE, unknown lipid P440, and unknown lipid P395.

A total of 606 lipids (27% of total metabolites) were detected and quantified in the samples derived from the two bacteria; however, majority were unknown (Fig. 5a). A detailed examination of the identified lipids revealed a variation in the relative levels of 14 lipid classes between the two strains (Table 4, Fig. 5b). The total of the minor components was greater in P4A, with ceramide (CER) and lysophosphatidylethanolamine (LPE) present at very low levels and monogalactosyldiacylglycerol (MGDG) being higher in P4A, while LPE appears below detection in 1045. The relative levels of phosphatidic acid (PA), phosphatidylglycerol (PG) and phosphatidylserine (PS) were higher in the P4A, whereas phosphatidylcholine oleoyl/palmitoyl (PC_o/p) and phosphatidylinositol (PI) were much lower to the levels in 1045 (Fig. 5b). This suggests there was a difference in lipid metabolism and biosynthesis between these two strains. Six different metabolites that exhibited significant differences in *P. oleovorans* P4A relative to 1045 include 4 known lipids (DG, PC, PE, and PS) and 2 unknown lipids (P440 and P395) that showed the greatest difference between the strains (Fig. 5c; Supplementary Table S4).

**Table 4.** The important known lipid concentrations in *P. oleovorans* strains 1045 and P4A

Lipids name*	1045	P4A	*t*-tests	*P*-value	FDR
CAR	5.6680	3.6587	1.1861	0.2800	0.2804
CER	0.4356	2.5627	−2.0831	0.0820	0.1190
DG	4.4042	11.0134	−2.7351	0.0340	0.1040
DG2	5.0341	8.8168	−1.3482	0.2260	0.2437
FAHFA	2.2143	7.0216	−2.4173	0.0520	0.1040
LPE	0.1995	1.0638	−2.0604	0.0850	0.1190
MGDG	2.0308	12.5461	−1.8183	0.1190	0.1513
PA	15.2806	42.2612	−3.2421	0.0180	0.1040
PC	31.3920	58.8857	−2.3973	0.0530	0.1040
PC_o/p	38.3427	7.4066	3.5837	0.0120	0.1040
PE	30.3228	56.7279	−1.4973	0.1850	0.2158
PG	14.0874	32.9320	-2.3201	0.0590	0.1040
PI	9.9297	3.4200	2.3833	0.0550	0.1040
PS	16.0737	40.1582	-2.6396	0.0390	0.1040

* Carnitine (CAR), ceramide (CER), diacylglycerol (DG), 1,2-diacylglycerol (DG2), fatty acid hydroxy fatty acid (FAHFA), lysophosphatidylethanolamine (LPE), monogalactosyldiacylglycerol (MGDG), phosphatidic acid (PA), phosphatidylcholine (PC), phosphatidylcholine oleoyl/palmitoyl (PC_o/p), phosphatidylethanolamine (PE), phosphatidylglycerol (PG), phosphatidylinositol (PI), and phosphatidylserine (PS).

## Discussion

This study identified and characterized the biocide-resistant microbe that was most probably responsible for the spoilage of industrial water-based coating materials. *Pseudomonas oleovorans* P4A was isolated from contaminated coating material containing isothiazolone preservatives and compared to a reference strain 1045, that was isolated from industrial fluids. *Pseudomonas oleovorans* is a Gram-negative, non-spore-forming bacterium known for its high versatility and intrinsic antibiotic resistance. It is a methylotrophic bacterium that can degrade organic compounds and can grow in diverse industrial matrices (Weiser et al., 2019). Despite it being a problematic contaminant in industry, *P. oleovorans* is known for its ability to degrade a broad range of hydrocarbons, making it valuable for the bioremediation of contaminated sites (Ma et al., 2012). Since *P. oleovorans* P4A was consistently isolated from spoiled coating materials containing isothiazolone biocides, it was considered to have a significant role in product spoilage.

The colony and cellular morphological differences observed between *P. oleovorans* P4A isolated from biocide containing water-based coating and 1045 isolated from biocide-free industrial cutting fluid may be due to adaptation to different environmental conditions and biocide exposure. Zhang and colleagues (Zhang et al., 2015) reported morphological diversity among *Pseudomonas* ssp isolated from different environments. The present results reflect previous studies that observed a relationship between microbial morphology and resistance mechanisms in *Pseudomonas* spp (Russell, 2003; Wang et al., 2023). Alterations in the cell wall or extracellular polysaccharide may result in different degrees of resistance to biocides. Other mechanisms that confer resistance to biocides are increased presence of efflux pumps, which expel toxic substances from the cells, and the formation of biofilms. Lyon et al. (2022) demonstrated that adaptive strategies, such as biofilm formation or the production of protective layers influence biocidal tolerance.

*Pseudomonas oleovorans* P4A had a slower growth rate and longer lag phase than the reference strain and this could be a contributing factor to biocide resistance. Previous studies on *Burkholderia*, a close relative of *Pseudomonas* spp, showed that isothiazolone-resistant strains had both lower growth rates and an extended lag phase (Rushton et al., 2013). Microbial growth rates are closely related to susceptibility to biocides, where a slower growth rate will reduce the impact of metabolic inhibition, and slower-growing populations exhibit increased resilience to antimicrobial agents (Russell, 2003). In the present study, we showed that *P. oleovorans* P4A was more resistant to biocides generally used for in-can preservation of water-based coatings than the reference strain 1045. The industrial isolate exhibited a 2.5-fold and 1.5-fold higher MIC value for CMIT and BIT, respectively, compared to *P. oleovorans* 1045 whereas, it displayed a lower MIC value for MIT. Maillard (2018) recently reviewed the mechanisms of biocide resistance in bacteria and identified that both detoxification and repair mechanisms were essential. Detoxification mechanisms include cell wall and membrane properties that reducing biocide penetration, efflux pumps, and enzymatic degradation. Reduced biocide activity has been associated with the lipopolysaccharide of Gram-negative cell wall, protein and membrane phospholipid and fatty acid content (Maillard, 2002). The mechanism of activity of isothiazolone biocides involves an initial stage that rapidly inhibits growth followed by a second stage that causes cell death after several hours (Williams, 2006). The initial stage disrupts metabolic pathways by blocking energy metabolism and adenosine triphosphate (ATP) synthesis and the second stage inactivates proteins by destroying di-sulphide bonds and producing free- radicals (Williams, 2006).

A correlation between bacterial resistance to biocides and resistance to antibiotics has previously been observed; however, this was not the case in the present study (Chen et al., 2021; Coombs et al., 2023). Both strains were sensitive to the antibiotics tested, showing no cross-resistance. *Pseudomonas oleovorans* P4A exhibited slightly higher MIC than 1045 to imipenem and meropenem; however, the MICs were lower for the remaining five antibiotics. Ceftazidime, a third-generation cephalosporin used for *Pseudomonas aeruginosa* infections, showed to be effective against both strains, while meropenem displayed a notable difference. Biocide-resistant *P. oleovorans* P4A displayed reduced tolerance to colistin, an antibiotic that disrupts bacterial cell membranes, and both aminoglycoside antibiotics (amikacin, tobramycin) tested compared to *P. oleovorans* 1045. Similarly, a *P. oleovorans* isolated from industrial wastewater containing high levels of sulfate compounds was also susceptible to a diverse array of antibiotics, including among others, imipenem, meropenem, ceftazidime, ciprofloxacin, tobramycin, and amikacin (Wang et al., 2023). These findings suggest that *P. oleovorans* resistance to toxic substances may not affect their sensitivity to antibiotics. Cross-resistance is attributed to bacteria using similar resistance mechanisms to biocides and antibiotics such as altered efflux pumps and/or uptake mechanisms, thus our results suggest that is not the case in P4A (Poole, 2002; Chapman, 2003; Zhou et al., 2015). The susceptibility of *P. oleovorans* to antibiotics targeting cell wall synthesis, such as imipenem and meropenem, demonstrated variations between the strains, indicating strain-dependent tolerance (Jorgensen & Ferraro, 2009). It is therefore likely that the mechanism of resistance to isothiazolone biocides does not influence the resistance to antibiotics.

*Pseudomonas* species are known to have intrinsic resistance to antimicrobial substances by the nature of the lipopolysaccharides and other lipids in the outer membrane. Phospholipid and fatty acid changes in the bacterial membrane have been implicated in increased resistance to biocides (Herndon et al., 2020). Our previous study had shown that *P. oleovorans* 1045 had to make greater metabolic alterations in lipid, amino acid, and energy metabolism than P4A, suggesting that P4A was already adapted to biocide stress (Ali et al., 2024). This prompted the present comparison of the lipidomic profiles between *P. oleovorans* P4A and *P. oleovorans* 1045. There were significantly higher levels of lipidic components in the biocide resistant P4A strain, while the polar metabolites were higher in 1045 (Fig. 4c). This was also visible in the relative concentration of several lipid classes (Table 4). These differences can be explained in part by alterations in lipid metabolism and biosynthesis between strains and may have a connection to the increased tolerance to biocides. Within the context of biocide resistance, specific lipid classes can play crucial roles.

Phosphatidic acid (PA), phosphatidylcholine oleoyl/palmitoyl (PC_o/p), phosphatidylserine (PS), phosphatidylcholine (PC), glycerolipids (DG), phosphatidylethanolamine (PE), and phosphatidylinositol (PI) are components involved in various cellular processes, and have been implicated in biocide resistance in *P. oleovorans* (Zhang & Rock, 2008; Wang et al., 2006). An association between higher levels of phosphatidic acid (PA) and increasd biocide resistance has been demonstrated (Yao & Rock, 2013; Sutterlin et al., 2014). In *P. oleovorans*, PA is involved in cellular processes, including cell signaling, membrane structure, and energy production (Craig et al., 2021). Studies show that increased PA levels may impact membrane fluidity that can create a permeability barrier and this would reduce biocide penetration into the bacterium (Sutterlin et al., 2014). Phosphatidic acid (PA), a key lipid molecule, has also been implicated in shielding bacterial cells from the impact of biocides. When exposed to biocides, PA accumulates and stabilizes the cell membrane, potentially forming a protective layer that reduces biocide penetration, and enhances cell survival. Recent studies suggest that PA may also participate in activating efflux pumps, which actively remove biocides from the cell (Adkin et al., 2022). Understanding the interaction between PA and bacterial defence mechanisms is necessary for controling biocide resistance effectively.

Strain P4A exhibited elevated levels of phosphatidylethanolamine (PE) and phosphatidylcholine (PC), suggesting modifications in membrane fluidity and biocide interactions that may influence resistance mechanisms (Xu et al., 2023). Additionally, the increased presence of phosphatidic acid (PA) in P4A implies an enhanced membrane remodeling response to biocide exposure, which may contribute to its overall resilience (Bernal et al., 2018). The reduction in oxidized phosphatidylcholine (PC_o/p) levels in P4A suggests a potential advantage in oxidative stress management, further supporting its classification as the more resistant strain. The biocide resistant *P. oleovorans* P4A had a much lower relative level of PC_o/p than 1045 reference strain. The cells with reduced PC_o/p levels exhibited heightened resistance to benzalkonium chloride suggesting a potential link between lower PC_o/p levels and decreased membrane fluidity, thus creating a barrier to biocide permeability (Barros et al., 2022). Targeting mechanisms that influence PC_o/p levels could present a promising avenue for enhancing the efficacy of biocides against resistant strains. The variations in phosphatidylinositol (PI) and phosphatidylglycerol (PG) levels between the two strains likely play a role in altering cellular signaling pathways and membrane charge, thereby influencing biocide susceptibility (Roth & Sternweis, 1997). Furthermore, the increased abundance of glycolipids such as MGDG (monogalactosyldiacylglycerol) in P4A may contribute to enhanced membrane stability, conferring an adaptive advantage under biocidal stress conditions (Vogel et al., 1998; Nomura et al., 2022). These lipid-mediated modifications, in conjunction with metabolomic adaptations, provide mechanistic insights into the differential responses of P4A and 1045 to CMIT, MIT, and BIT.

The presence of phosphatidylethanolamine (PE), phosphatidylglycerol (PG), and phosphatidylinositol (PI) may play a role in bacterial resistance (Wilderman et al., 2002). PI is a phospholipid that is involved in various cellular processes, such as membrane trafficking, signal transduction, and cytoskeleton organization (Constantino-Teles et al., 2022). Previous studies reported the role of phosphatidylinositol in regulating membrane fluidity and permeability of *Pseudomonas* bacteria (Balla, 2013; Verdial et al., 2023). This finding supports the idea that biocide resistance in *Pseudomonas spp* may involve membrane functions such as efflux pumps, changes in membrane properties, in addition to enzymatic breakdown and biofilm formation (Namaki et al., 2022).

Diacylglycerol (DG) and 1,2-diacylglycerol (DG2) were slightly increased in the biocide resistant *P. oleovorans* P4A strain. DGs in bacterial cell membranes extend beyond mere structural support, influencing susceptibility to biocides, especially those targeting cell wall functions (Murinova & Dercova, 2014). Their contribution to membrane properties, including fluidity, permeability, and stability, has been well-documented (Goni & Alonso, 1999). Variations in DG levels are associated with changes in cell wall attributes, implying its participation in maintaining the integrity of the cell envelope (Carrasco & Merida, 2007).

Consistent with our results, an increase in the concentration of PS in *P. oleovorans* bacteria exposed to biocides compared to their unexposed counterparts was previously observed (Sun et al., 2014). This raises questions about the potential influence of increasing PS levels on the bacteria’s ability to resist biocides. The lipid composition of bacterial membranes is known to influence their permeability and resistance to external stressors including biocides (Willdigg & Helmann, 2021). Increasing PS levels may alter the structural properties of the bacterial membrane affecting its ability to repel or neutralize biocidal agents. Furthermore, PS has been implicated in signal transduction pathways suggesting a potential role in modulating gene expression related to biocide resistance mechanisms. Willdigg & Helmann (2021) reported that *Bacillus subtilis* increase the levels of PS in response to stressors such as heat, ethanol and acidity. Change in PS levels may alter the bacterial membrane’s structure, affecting its ability to repel biocidal agents. Moreover, PS is implicated in signaling pathways, potentially influencing gene expression related to biocide resistance. *Bacillus subtilis* adapts its membrane composition to different stressors targeting the cell envelope, aiding its survival and adaptation (Willdigg & Helmann, 2021). A previous study demonstrated that PS is required for the optimal function of the multidrug efflux pump, BmrA in *B. subtilis*, which confers resistance to various antibiotics and biocides (Willdigg & Helmann, 2021). In addition, genes mexAB-oprM, mexCD-oprJ, and mexEF-oprN, found in *P. aeruginosa*, are known regulators of efflux pumps linked to the tolerance to biocides (Verdial et al., 2023). This demonstrates that changes in PS levels in bacterial membranes can influence the expression of genes associated with biocide resistance, providing a potential mechanistic link between lipid composition and resistance phenotypes.

In summary, the industrial isolate of *P. oleovorans* has cellular, morphological, and physiological changes that may be part of the adaptive features leding to high levels of CMIT and BIT biocide tolerance. Understanding these mechanisms may contribute to the effective, but responsible use of biocides and the development of new effective strategies in managing microbial contamination in industry.

## Supplementary Material

kuaf015_Supplementary_File

## Data Availability

The data underlying this article that is not in the online supplementary material will be shared on reasonable request to the corresponding author.
