# Supplementary material for: Biocide-resistant Pseudomonas oleovorans isolated from water-based coatings used in construction
Source: J Ind Microbiol Biotechnol. 2025 Jun 17;52:kuaf015. doi: 10.1093/jimb/kuaf015 (PMC12231568; doi:10.1093/jimb/kuaf015)
Supplement: kuaf015_Supplementary_File [file kuaf015_supplementary_file.pdf]

Supplementary material

Biocide-resistant *Pseudomonas oleovorans* isolated from water-based coatings used in construction.

Muatasem Latif Ali[1,2], Lionel Ferrieres[3], Tuulia Hyötyläinen[1], Jana Jass[4]*

[1] MTM, School of Science and Technology, Örebro University, Sweden

[2] Saint-Gobain Sweden AB, Scanspac. Sweden

[3] Saint-Gobain Research Paris, Aubervilliers, France

[4] The Life Science Center-Biology, School of Science and Technology, Örebro University, Sweden

* Correspondence: Jana Jass, The Life Science Center -Biology, School of Science and Technology, Örebro University, Fakultetsgatan 1, 701 82 Örebro, SWEDEN.

Email: jana.jass@oru.se

Supplementary Table S1. The general set-up of the 96-well plate for the MIC test.

No.	5-chloro-2-methyl-4-isothiazolin-3-one (CMIT) (mg/L)	Benzothiazolinone (BIT) (mg/L)	2-Methyl-3(2H)-isothiazolinone (MIT) (mg/L)
1	2	300	20
2	4	400	25
3	6	500	30
4	8	600	35
5	10	700	40
6	12	800	45
7	14	900	50
8	16	1000	55
9	18	1000	60
10	20	1200	65
11	22	1300	70
12	24	1400	75

Supplementary Table S2. Internal standards added to the extraction solution.

Compound	Fc	Stock (ppm)	Volume (µL)
Hexanoic acid-d3 (MeOH)	10	1000	750
Heptadecanoic acid	10	1000	750
Betaine-d11	5	1000	375
Tryptophan (indole)-d5	30	2000	1.125
Valine-d8	20	1000	1.5
Indole-d6	23.33	1000	1.75
Benzoic acid-d5	13.33	1000	1
Succinic acid-d4	20	1000	1.5
Taurocholic acid (TCA)-d4	1	1000	75
Lithocholic acid (LCA)-d4	1	1000	75
Glycolithocholic acid (GLCA)-d4	1	1000	75
Glycoursodeoxycholic acid (GUDCA)-d4	1	1000	75
Deoxycholic acid (DCA)-d4	1	1000	75
Glycochenodeoxycholic acid (GDCA)-d4	1	1000	75
Glycocholic acid (GCA)-d4	1	1000	75
Cholic acid (CA)-d4	1	1000	75
Ursodeoxycholic acid (UDCA)-d4	1	1000	75
M8PFOA-13C8	0.2	50	300
MPFNA-13C5	0.2	50	300
M7PFUnDA-13C7	0.2	50	300
M3PFHxS-13C3	0.2	50	300
M8PFOS-13C8	0.2	50	300

Supplementary Table S3. Compounds used in calibration curves and internal standard mixtures for lipidomic analysis.

**Calibration standards**	**Internal standards**
CE (18:0)	Cer (d18:1/17:0)
CE (18:2)	LPC (17:0)
Cer(d18:0/18:1(9Z))	PC (16:0/d31/18:1)
DG (18:0/18:2)	PC (17:0/17:0)
LPC (16:0)	PE (17:0/17:0)
LPC (18:0)	SM(d18:1/17:0)
LPC (18:1)	TG (17:0/17:0/17:0)
LPE (18:1)	
PC (16:0e/18:1(9Z)	
PC (18:0p/18:1(9Z)	
PC (18:0p/22:6)	
PE (16:0/18:1)	
PE (16:0/18:1(9Z)	
TG (16:0/16:0/16:0)	
TG (18:0/18:0/18:0)	

Supplementary Table S4. Comparison of lipid profiles in *P. oleovorans* 1045 reference and P4A isolate selected by t-test ($p < 0.05$).

Unknown Lipid ID	t-test	p-value	FDR[1]
619	-8.5500	0.0001	0.0850
1202	6.8800	0.0005	0.1075
1751	-6.3800	0.0007	0.1075
1375	-6.3600	0.0007	0.1075
433	5.4900	0.0015	0.1594
1396	5.4600	0.0016	0.1594
762	4.9300	0.0026	0.2267
744	-4.5400	0.0040	0.2698
1495	-4.5200	0.0040	0.2698
841	4.0400	0.0068	0.3133
1132	-3.9000	0.0080	0.3133
976	-3.8700	0.0083	0.3133
1390	3.8500	0.0084	0.3133
1393	3.6600	0.0106	0.3133
924	3.6000	0.0113	0.3133
1387	3.5900	0.0115	0.3133
788	3.5600	0.0119	0.3133
1041	-3.4700	0.0133	0.3133
1485	-3.4700	0.0133	0.3133
1388	3.4100	0.0143	0.3133
709	3.3300	0.0159	0.3133
1386	3.2900	0.0166	0.3133
1648	-3.2800	0.0169	0.3133
835	-3.2300	0.0179	0.3133
607	-3.2300	0.0180	0.3133

907	-3.2100	0.0183	0.3133
543	3.2100	0.0183	0.3133
1068	-3.1900	0.0188	0.3133
1073	-3.1500	0.0197	0.3133
1191	-3.1200	0.0205	0.3133
403	-3.1000	0.0212	0.3133
961	-3.0500	0.0224	0.3133
1622	-3.0500	0.0225	0.3133
1798	-3.0100	0.0236	0.3133
1043	-2.9800	0.0246	0.3133
1501	-2.9700	0.0250	0.3133
1156	-2.9200	0.0266	0.3133
808	-2.9200	0.0266	0.3133
1685	-2.9100	0.0268	0.3133
24	2.8700	0.0283	0.3133
963	-2.8300	0.0299	0.3133
1262	-2.8200	0.0303	0.3133
1313	-2.8000	0.0311	0.3133
1757	-2.8000	0.0313	0.3133
949	-2.8000	0.0313	0.3133
245	-2.7900	0.0317	0.3133
1295	-2.7800	0.0322	0.3133
1744	-2.7600	0.0328	0.3133
1145	-2.7500	0.0334	0.3133
1193	-2.7500	0.0335	0.3133
1807	-2.7400	0.0336	0.3133
005	-2.7400	0.0336	0.3133
1160	-2.7400	0.0338	0.3133
1157	-2.7200	0.0344	0.3133

1756	-2.7200	0.0344	0.3133
1801	-2.7200	0.0348	0.3133
1243	-2.7000	0.0357	0.3133
799	-2.7000	0.0358	0.3133
1299	-2.6700	0.0372	0.3133
303	-2.6700	0.0373	0.3133
1173	-2.6600	0.0373	0.3133
1251	-2.6500	0.0378	0.3133
1106	-2.6500	0.0380	0.3133
1728	-2.6500	0.0381	0.3133
1260	-2.6400	0.0385	0.3133
1085	-2.6400	0.0387	0.3133

[1] FDR: False discovery rate.

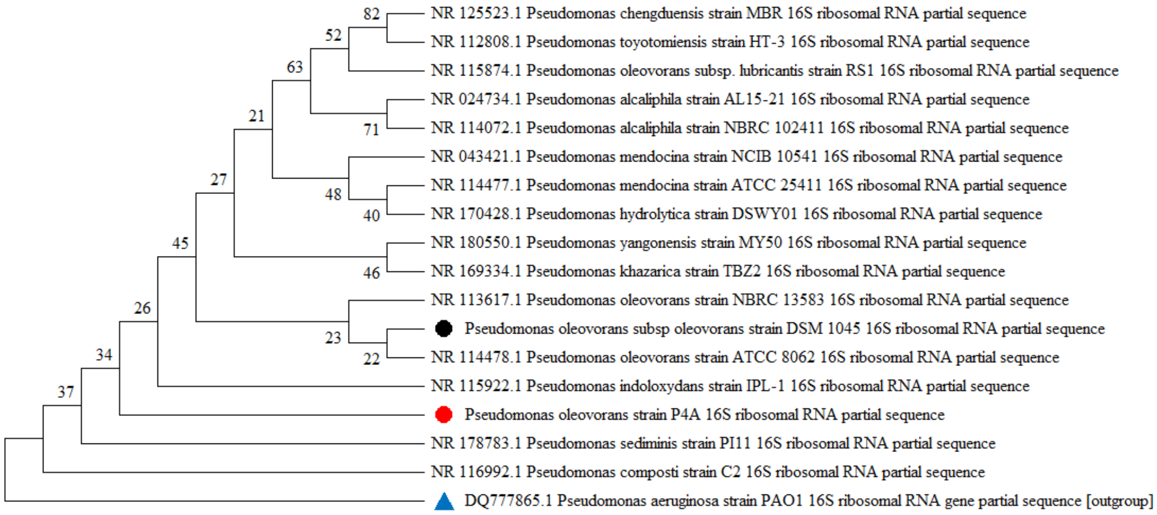

Supplementary figure S1. Evolutionary relationship between *P. oleovorans* P4A and *P. oleovorans* P1045 is shown with a phylogenetic tree inferred using maximum likelihood method. The tree is constructed with an outgroup (shown in blue traingle). Using BLAST analysis, the two strains have 99.93% similarity. *P. oleovorans* P4A isolate was sent to Eurofins Genomics (Germany) for sequencing for 16S rRNA analysis.
